# Synthesis of Porous Polymers by Nucleophilic Substitution Reaction of Polyamines and Monochlorotriazinyl-β-Cyclodextrin and Application to Dye Adsorption

**DOI:** 10.3390/ma18112588

**Published:** 2025-06-01

**Authors:** Naofumi Naga, Risa Hiura, Tamaki Nakano

**Affiliations:** 1 College of Engineering, Shibaura Institute of Technology, 3-7-5 Toyosu, Koto-ku, Tokyo 135-8548, Japan; 2 Graduate School of Science & Engineering, Shibaura Institute of Technology, 3-7-5 Toyosu, Koto-ku, Tokyo 135-8548, Japan; ad20079@shibaura-it.ac.jp; 3 Institute for Catalysis and Graduate School of Chemical Sciences and Engineering, Hokkaido University, N 21, W 10, Kita-ku, Sapporo 001-0021, Japan; tamaki.nakano@cat.hokudai.ac.jp; 4 Integrated Research Consortium on Chemical Sciences, Institute for Catalysis, Hokkaido University, N 21, W 10, Kita-ku, Sapporo 001-0021, Japan

**Keywords:** polyamine, β-cyclodextrin, chlorotriazine, nucleophilic substitution reaction, phase separation, porous polymer, dye adsorption

## Abstract

Network polymers with β-cyclodextrin moieties were prepared by nucleophilic substitution reactions between polyamines, linear polyethyleneimine (LPEI), polyallylamine (PAA), (ε-poly-L-lysine) (EPL), and monochlorotriazinyl-β-cyclodextrin (MCTCD) in methanol/water mixed solvent or water. The reactions under conditions of high material concentration (30 wt%) and a feed ratio of [MCT]/[NH] = 0.5 (mol/mol) successfully yield porous polymers via reaction-induced phase separation. The molecular structure of the polyamines and reaction conditions strongly affected the morphology of the resulting porous polymers. The porous polymers were composed of connected particles, gathered (slightly connected) particles, and/or disordered bulky structures, with sizes of 10^−9^ m–10^−8^ m. An increase in the molecular weight of LPEI and PAA and the feed molar ratio of [MCT]/[NH] tended to decrease the particle size. Young’s moduli of the LPEI-MCTCD and PAA-MCTCD porous polymers increased with an increase in bulk density, which was derived from small particle sizes. The wide particle size distribution and disordered structure caused collapse by the compression under 50 N of pressure. An LPEI-MCTCD adsorbed methyl orange, methylene blue, and phenolphthalein through ionic interactions, π–π interaction, and/or β-cyclodextrin inclusion.

## 1. Introduction

Amine-based polymers, which contain amino groups either in the main chain or as side groups, have been widely used due to their distinctive properties. For example, poly(acrylamide) is used as flocculants for water treatment, paper-strengthening agents, and so on. Poly(N-isopropylacrylamide) is known as a functional polymer, which shows thermal responsibility. Polyethyleneimine (PEI) is widely used as a laminate anchor coating agent, heavy metal-chelating agent, metal plating additive, adhesive, water treatment, etc. [1,2,3,4,5,6,7,8,9,10]. PEI is commercially produced by the ring-opening polymerization of ethyleneimine and has a branching structure with primary, secondary, and tertiary amine groups [11]. Polyallylamine (PAA) is also a commercially produced amine-based polymer synthesized by polymerization of allylamine. PAA-based polymers have individual functionalities derived from amine groups in the side chain [12,13,14,15,16]. Further applications of PAA are possible by using the high reactivity of the amine groups. The crosslinking reaction of the polyamines should be suitable for producing the network polymers. Various reactions are applicable for the crosslinking reaction due to the high reactivity of the primary and secondary amine groups. For example, polyurea is prepared by an additional reaction of polyamine and diisocyanate. The ring-opening addition reaction of multi-functional epoxy and amine compounds yields epoxy resin. The Aza-Michael nucleophilic addition reaction between amine and acrylate groups is also useful for preparing the network polymers [17,18,19,20].

**Scheme 1:**
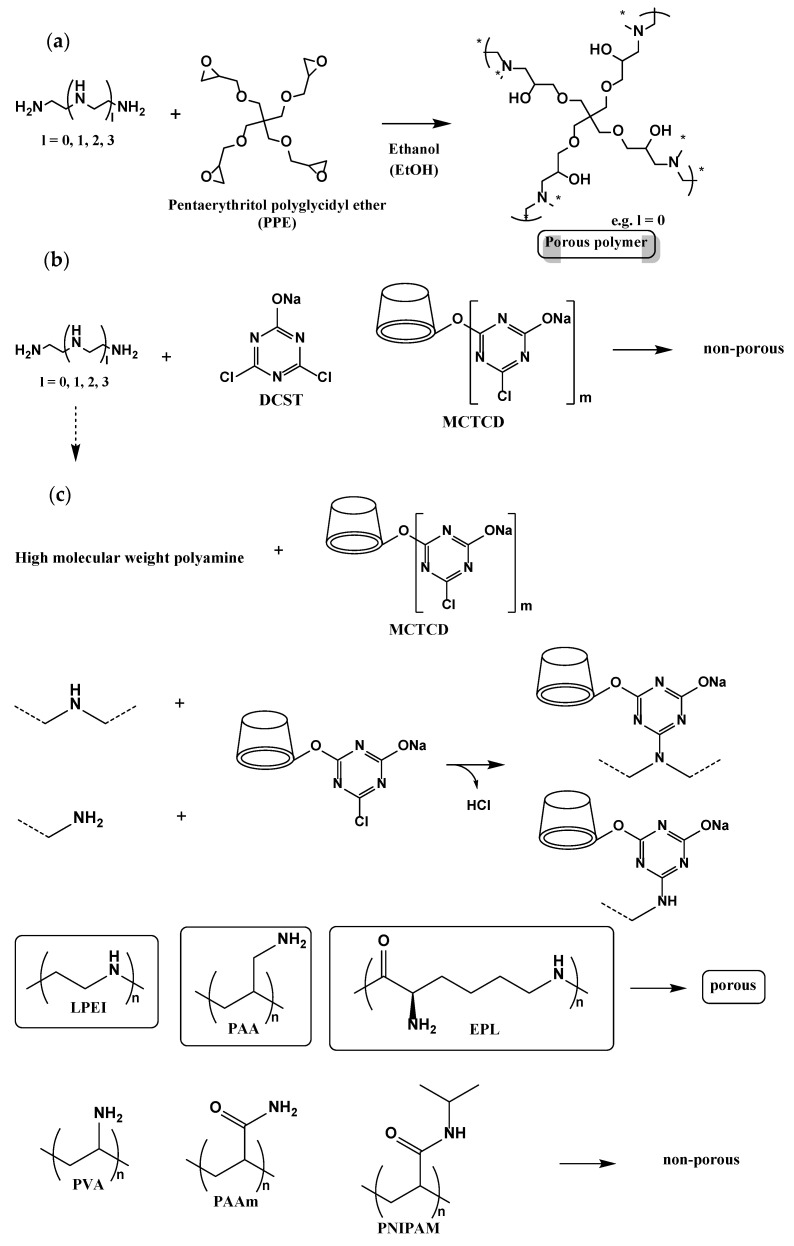
An outline of the research: (**a**) a previous study, (**b**) a preliminary study, and (**c**) this study, where porous polymers are prepared through the addition reactions of polyamines.

Chlorotriazine shows high reactivity with the amine group. Addition reactions between dichlorotriazine and diamine molecules yield linear and network polymers [21,22,23]. Crosslinking reactions of amine-based polymers with multi-functional chlorotriazine molecules, such as monochlorotriazinyl-β-cyclodextrin (MCTCD), represent an effective approach to forming network polymers. Uyama and co-workers reported the synthesis of monolithic porous polymers via an addition reaction of MCTCD and PEI in water [24]. The resulting monolithic polymers adsorbed various dyes through encapsulation with the β-cyclodextrin (β-CD) moiety in MCTCD. Their findings suggest the potential of MCTCD as a multi-functional crosslinking molecule with polyamines. Recently, we reported ring-opening addition reactions between low molecular weight linear polyethylene amine, such as ethylenediamine, diethylenetriamine, triethylenetetramine, tetraethylenepentamine, and a tetra-functional epoxy compound, pentaerythritol polyglycidyl ether, to yield network polymers, including porous polymers induced by phase separation (Scheme 1a) [25]. We have investigated the preparation of the porous polymers through a series of considerations. We applied multi-functional chlorotriazine molecules, 4,6-dichloro-2-sodiooxy-1,3,5-triazine (DCST) and MCTCD, on behalf of the tetra-functional epoxy, as crosslinkers of these polyethylene amine molecules (Scheme 1b). Although the network polymers were obtained by the reactions between the low molecular weight linear polyethylene amine and DCST or MCTCD, porous polymers were not produced. We found that reactions between high molecular weight linear polyethyleneimine (LPEI) and MCTCD in methanol/water mixed solvent could produce porous polymers. We are developing porous polymers with CD moieties using several methods [26]. Judging from our results, a combination of CD molecules and polymer materials is important to control the mechanical and physical properties of the resulting porous polymers. Variations of polymer materials should expand the application possibilities. We have investigated the preparations of the porous polymers with CD moieties using a series of polyamines, including amine groups in the main chains or in the side chains. In this paper, we report the nucleophilic substitution reaction between several polyamines and MCTCD to yield the porous polymers (Scheme 1c). We particularly focus on the effect of the molecular structure of these polyamines on the state of the resulting polymers and the morphology and mechanical properties of the porous polymers.

The resulting porous polymers with MCTCD have some characteristic moieties in the polymer networks, tertiary amine, triazine, and β-CD, whose structures should be applicable for adsorption of molecules from solutions. The β-CD moiety can incorporate organic compounds via a host–guest mechanism. The positive-charged surface derived from tertiary amine groups can absorb the molecules with a negative charge. Furthermore, triazine moiety derived from MCTCD may adsorb aromatic molecules through π–π interactions. As model cases, we investigate the adsorption of methyl orange, methylene blue, and phenolphthalein by an LPEI-MCTCD porous polymer.

## 2. Materials and Methods

### 2.1. Materials

Monochlortriazingl-β-cyclodextrin (MCTCD) was kindly donated by CycloChem Bio Co., Ltd. (Kobe, Japan), and used as received. Polyallylamine (PAA) aq. solutions (PAAX: Y wt%, X = weight average molecular weight [M_w_] of PAA, Y = concentration of PAA in solution, PAA1600: 15 wt%, PAA3000: 20 wt%, PAA5000: 20 wt%, PAA8000: 15 wt%) were kindly donated by Nittobo Medical Co., Ltd. (Tokyo, Japan), and used as received. ε-Poly-L-lysine (EPL) was kindly donated by Okuno Chemical Industries Co., Ltd. (Osaka, Japan), and used as received. Poly(2-ethyl-2-oxazoline) (PEOX) samples with different molecular weights (Mw: 50,000, 500,000) were commercially obtained from Sigma-Aldrich, Co. LCC, St. Louis, MO, USA), and used as received. N-Vinylformamide (Tokyo Chemical Industry Co., Ltd. Tokyo, Japan) and acrylamide (Fuji-film Wako Pure Chemical Industries, Osaka, Japan) were commercially obtained and were purified by activated Al_2_O_3_ to remove inhibitors before use. Ammonium persulfate (APS) (Fuji-film Wako Pure Chemical Industries) and N,N,N′,N′-tetramethylethylenediamine (TEMED) (Tokyo Chemical Industry Co., Ltd.) were commercially obtained and used as received. Methanol (MeOH), ethanol (EtOH), NH_3_ aq. (20 wt%), methyl orange (MO), methylene blue (MB), and phenol phthalein (PP) were commercially obtained from Kanto Chemical Co., Inc. (Tokyo, Japan), and used without further purification.

LPEI was prepared according to the literature, as described below [27]. PEOX (5.94 g, Mw: 50,000 or 500,000), and HCl aq. (6.0 mol/L), 25.0 mL) were added to a 200 mL two-necked flask equipped with a condenser and a stirrer piece. After purging N_2_ for 1 h, the reaction system was heated for 9 h at 90 °C with stirring. After cooling to room temperature, NH_3_ aq. (25 wt%) was added to the reaction solution until the pH reached 7.0. The reaction solution was poured into a Petri dish and roughly dried in the fume hood under the atmospheric conditions for 24 h. The resulting LPEI was purified by dialysis in NH_3_ aq. using dialysis tube (visking tubing: 19.1 mmϕ * 250 mm, hole diameter: 50 Å) for 72 h. The NH_3_ aq. was replaced once a day. The purified LPEI solution was poured into a Petri dish and roughly dried in the fume hood under atmospheric conditions for 24 h, followed by drying under reduced pressure for 12 h. The LPEI prepared from PEOX with Mw of 50,000 or 500,000 was termed LPEI50K or LPEI500K, respectively.

Poly(vinyl amine) (PVA) was prepared through the hydrolysis of poly(N-vinylformamide) according to the literature [28]. Dichlorotriazine sodium salt (DCST) was synthesized according to the literature [29] and used as prepared aq. solution (20 wt%). Poly(acrylamide) (PAAm) or poly(N-isopropyl acrylamide) (PNIPAM) was synthesized by radical polymerization of acrylamide or N-isopropyl acrylamide in water using an APS/TEMED initiator system at room temperature and was used as a prepared aq. solution (20 wt%).

### 2.2. Preparation of Network Polymers

The reaction of LPEI50K-MCTCD at 30 wt% materials (LPEI50K + MCTCD), with [MCT]/[NH]: 0.5 (mol/mol), in a MeOH–water mixture (20/80 wt%/wt%) is described as an example. MCTCD (0.893 g), LPEI50K (0.16 g, 20 wt%), MeOH (0.70 g), and distilled water (2.8 g) were added to a quadrangular prism polyethylene bottle (1.7 cm × 1.7 cm × 3.0 cm), stirred using a vortex mixer (Mixer N-40M-1, NISSIN, Tokyo, Japan) for a few minutes to make a homogeneous solution, and then stored at room temperature for 2 h. The obtained porous polymer was washed by immersion in excess methanol for 24 h. The porous polymer composite was air-dried at room temperature for 24 h and further dried in vacuo at room temperature for 3 h. Reactions with LPEI500K, with varying [MCT]/[NH] ratios, materials concentrations, and solvent compositions, were conducted using the same procedures.

The reaction of PAA3000-MCTCD at a 20 wt% materials (PAA3000 + MCTCD) concentration, with an [MCT]/[NH]: 0.5 (mol/mol), in water is described as an example. MCTCD (0.893 g), PAA3000 solution (0.714 g, 20 wt%), and distilled water (2.417 g) were added to a quadrangular prism polyethylene bottle (1.7 cm × 1.7 cm × 3.0 cm), stirred with a vortex mixer for a few minutes to make a homogeneous solution, and then stored at room temperature for 2 h. The obtained porous polymer was washed through immersion in excess methanol for 24 h. The porous polymer composite was air-dried at room temperature for 24 h and further dried in vacuo at room temperature for 3 h. Reactions with other PAA, EPL, or different [MCT]/[NH] ratios and materials concentrations were conducted following the same procedures.

### 2.3. Adsorption Test of Dyes

MO, MB aq. solutions (1.0 mol/L) (neutrality), and a PP solution (0.03 mol/L, 20.0 g/L) in Na_2_CO_3_ aq. (alkalinity, pH: 11.3) were prepared for the adsorption test. A cubic sample of an LPEI-MCTCD porous polymer (0.8 × 0.8 cm × 0.8 cm) was soaked into 10 mL of the dyes’ solutions for 24 h at 25 °C.

### 2.4. Analytical Procedures

FT-IR spectra of porous polymers were recorded on an FTIR-8400 or an IRAffinity-1S (SHIMADZU Corporation, Kyoto, Japan), and 30 scans were accumulated from 4000 to 500 cm^−1^.

Scanning electron microscopy (SEM) images of Pt-coated porous polymers were acquired using a JSM-7610F microscope (JEOL, Tokyo, Japan) equipped with a low-energy ion detector at acceleration voltages of 3.0 kV or 20 kV, respectively. The sizes of particles in the SEM images were evaluated through image analysis using an Image-J software v 1.54 k package.

The surface area of the porous polymer was measured by nitrogen sorption using an Autosorb 6AG (Quantachrome Instruments, Boynton Beach, FL, USA) and a Belsorp II mini (MicrotracBEL Corp., Osaka, Japan). The samples were dried under reduced pressure at 100 °C for 1 h immediately before the measurement.

The mechanical properties of the porous polymer composites were investigated using compression tests conducted with a Tensilon RTE-1210 apparatus (ORIENTEC Co. Ltd., Tokyo, Japan). The test samples were cut into a 1 cm^3^ cube and pressed at a rate of 0.5 mm/min at room temperature. The bulk density of the porous polymer composite, g/cm^3^, was calculated from the weight of the samples before the test.

The UV-Vis spectra of the MO, MB, and PP solutions were recorded with a UV2450 spectrometer (SHIMADZU Corporation, Kyoto, Japan).

## 3. Results and Discussion

### 3.1. Synthesis of Network Polymers

Nucleophilic substitution reactions between LPEI50K, 500K, and MCTCD were conducted in the MeOH/water mixed solvents. Reactions in MeOH/water mixed solvents of 20/80 or 10/90 wt%/wt%, with a materials concentration of 30 wt% and an [MCT]/[NH] molar ratio of 0.5 or 1.0, successfully induced phase separation at room temperature to yield porous polymers regardless of the molecular weight of LPEI (Figure S1).

The PAA and MCTCD reactions were conducted in water at room temperature. The reactions with PAA1600 and PAA3000, at a materials concentration of 30 wt% and an [MCT]/[NH] molar ratio of 0.5 or 1.0, produced the corresponding porous polymers. In the corresponding reactions with a 20 wt% materials concentration, the porous polymers were obtained in the reactions with an [MCT]/[NH] molar ratio of 0.5. Although reactions with an equivalent molar ratio of [MCT]/[NH] (1.0) induced phase separation, the resulting polymers were fragile and collapsed easily. Reactions with PAA5000 and PAA8000 also induced phase separation but yielded fragile solid polymers (Figure S2).

The EPL and MCTCD reactions were conducted in water at room temperature. The reactions with a 30 wt% materials concentration and [MCT]/[NH] molar ratios of 0.5 or 1.0 preferentially produced porous polymers (Figure S3).

The PVA, PAAm, and PNIPAM reactions with MCTCD were conducted under various conditions. However, the corresponding porous polymers were not obtained. One explanation for these results is the high affinity between the amine polymers and solvent (water), which can be estimated by solubility parameters (SPs). The calculated SP values of PVA, PAAm, and PNIPAM are 12.1, 19.1, and 12.0 (cal./cm^3^)^1/2^ [30]. These values are higher than those of LPEI (10.9 (cal./cm^3^)^1/2^) and PAA (11.1 (cal./cm^3^)^1/2^). The small difference in SP values between the polymers and water (23.4 (cal./cm^3^)^1/2^) means higher affinity, which should decrease the phase separation rate in the reaction system and yield the gels. In addition, high viscosities in the PAAm and PNIPAM reaction systems would also hinder the phase separation.

In all reaction systems that produced porous polymers (LPEI, PAA, EPL-MCTCD), the reactions with a low [MCT]/[NH] molar ratio (0.1) resulted in gels or partially phase-separated gels. The high content of unreacted NH in the polymer networks caused by low crosslinking densities should not induce phase separation due to the high affinity between the polymer networks with the solvents used. The reactions with 20 wt% materials concentrations tended to yield the fragile solids. A higher materials concentration of 30 wt% is preferable for yielding porous polymers. One explanation for the result is that the polymer networks formed in reactions with low materials concentrations are insufficient to occupy the space of the reaction system.

Reactions with high molecular weights (Mw: 50 K, 500 K) of LPEI yielded porous polymers. By contrast, low molecular weight (Mw: 1600, 3000) should be favorable for producing PAA-MCTCD porous polymers. The difference is derived from the difference in the structure of the amine groups: secondary amine (NH in LPEI) versus primary amine (NH_2_ in PAA). Generally, the reactivity of primary amines is higher than that of secondary amines. In the case of reactions with PAA, high molecular weight PAA increases the viscosity of the reaction system, which decreases the reactivity of NH, causing the system to gel (precipitate) at lower NH conversion. As a result, low crosslinking density in the network induces fragile polymers.

### 3.2. Structure of Porous Polymers

**Figure 1:**
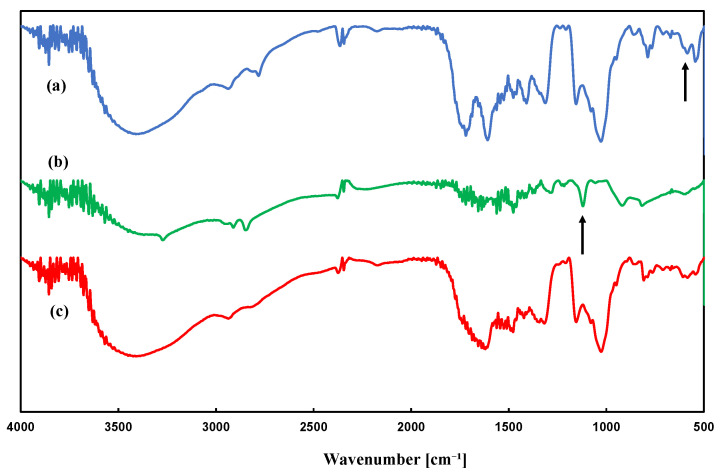
FT-IR spectra of the LPEI50K-MCTCD system: (**a**) MCTCD, (**b**) LPEI50K, and (**c**) LPEI50K-MCTCD, with an [MCT]/[NH] molar ratio of 0.5 and materials concentration of 30 wt%, in a MeOH/water mixed solvent at 10 wt%/90 wt% (entry 5).

The molecular structure of the porous polymers was qualitatively studied by FT-IR spectroscopy. Figure 1 shows the FT-IR spectra of LPEI, MCTCD, and LPEI-MCTCD porous polymers. The peaks derived from the triazine ring are detected at 1600 and 1720 cm^−1^. The peaks ranging from 1200–1500 cm^−1^ are attributed to the β-CD moiety. The intensity of an absorption peak at about 500–600 cm^−1^, derived from the Cl of the MCT moiety, was decreased in the resulting porous polymers [29]. Furthermore, the decrease in peak intensity was derived from the secondary amine group (1150–1110 cm^−1^) of LPEI. Similarly, the decrease in peak intensity derived from the primary amine groups of PAA (1150–1050 cm^−1^) and EPL (1640–1560 cm^−1^) was also observed in the porous polymer (Figures S4 and S5). These results indicate the progress of the reaction between the MCT and NH groups.

**Scheme 2:**
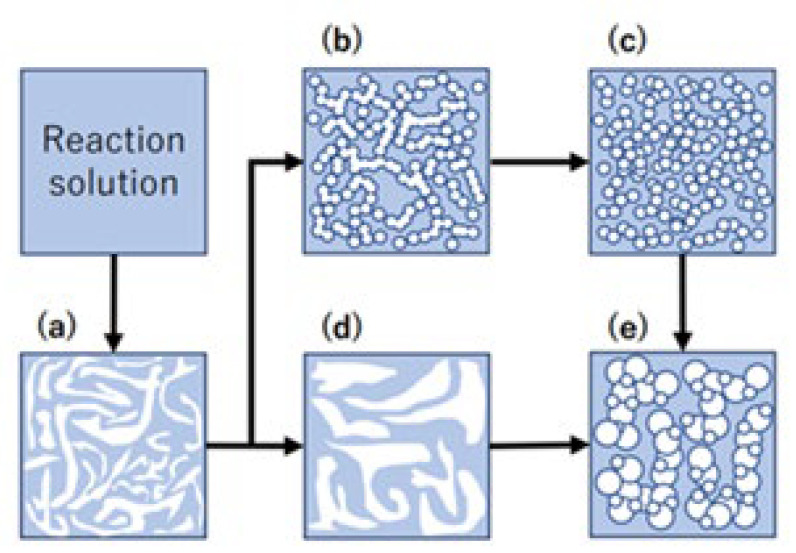
A model of polymerization-induced phase separation via spinodal decomposition process: (**a**) co-continuous structure at the early stage of phase separation, (**b**) transition state from co-continuous structure to small particles, (**c**) formation of small particles, (**d**) growth of the co-continuous structure, and (**e**) large particles derived from growth of the particles or grown co-continuous structure by interfacial tension, arrow means progress of the phase separation.

**Figure 2:**
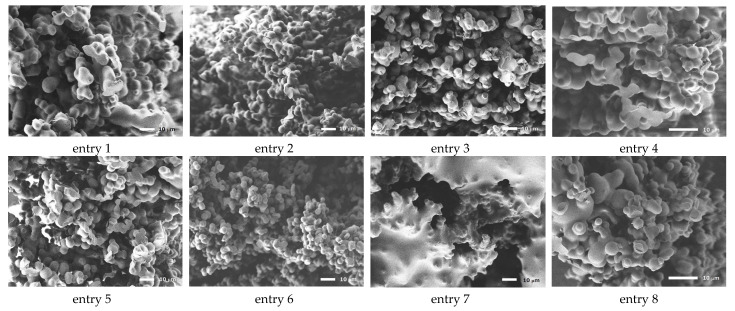
SEM images of LPEI-MCTCD porous polymers; scale bar = 10 μm.

**Figure 3:**
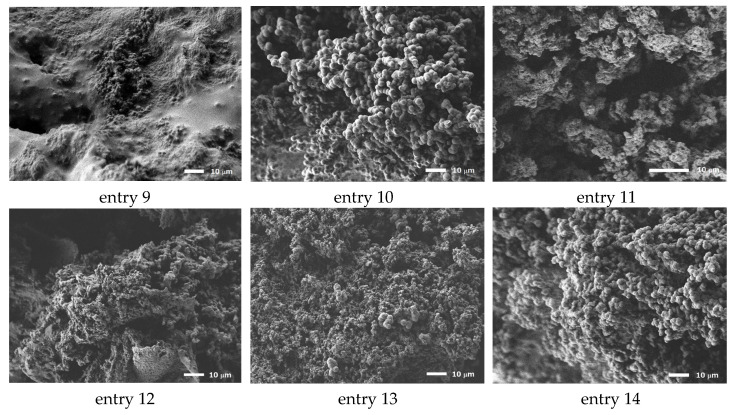
SEM images of PAA-MCTCD porous polymers; scale bar = 10 μm.

**Figure 4:**
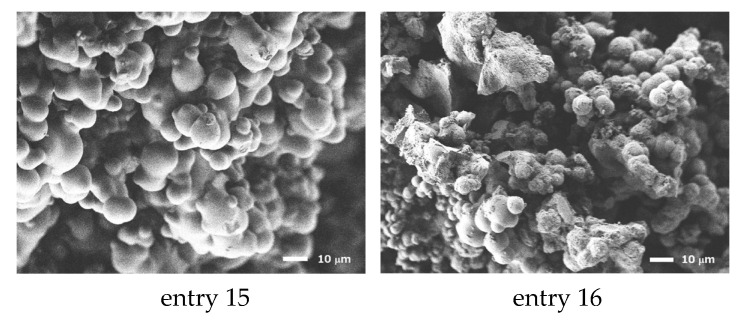
SEM images of EPL-MCTCD porous polymers; scale bar = 10 μm.

SEM images of representative LPEI, PAA, and EPL-MCTCD porous polymers are shown in Figure 2, Figure 3 and Figure 4. The surface morphology, particle size, and size distribution are summarized in Table 1. The porous polymers show surface morphologies composed of connected particles (C), gathered (contacted or slightly connected) particles (G), and/or disordered structures (D), at sizes of 10^−9^–10^−8^ m. These structures in the surface morphologies are likely formed by polymerization-induced phase separation via the spinodal decomposition process, as shown in Scheme 2. When the reaction system is fixed by network formation before phase separation, it yields a gel. In the early stage of phase separation, a co-contentious structure is formed (Scheme 2a,d). This structure transforms into particles by interfacial tension, accompanied by particle growth (Scheme 2b,c,e). The morphology of the porous polymer depends on the fixation stage of the phase separation, which should be determined by the relative ratio of the network formation rate and the phase separation rate. The structure of the porous polymers is characterized based on the size (diameter) distribution of the connected particles evaluated by image analysis of the SEM images.

The particle sizes of the LPEI-MCTCD porous polymers decreased with increasing molecular weight of LPEI (LPEI50K, LPEI500K) and with higher [MCT]/[NH] feed ratios, as shown in Figure 2 and summarized in Table 1. The increase in LPEI molecular weight increased the viscosity of the reaction systems, which likely decreased the phase separation rate. As a result, the morphology was fixed at the early stage of the phase separation. An increase in the [MCT]/[NH] feed ratio should effectively increase the network formation rate due to the higher crosslinking density in the polymer networks. Disordered bulky structures, likely resulting from incompletely phase separated parts, were observed in the porous polymer obtained under conditions with [MCT]/[NH] = 0.5 mol/mol, especially in the sample of entry 7. The composition of the MeOH/H_2_O mixed solvent used in the reactions to prepare the LPEI-MCTCD porous polymers also affected the morphology and particle size. An increase in H_2_O content in the mixed solvent would increase the network formation rate due to an increase in basic dissociation constant, thereby fixing the morphology with small particle sizes at the early stage of phase separation.

SEM images of the PAA-MCTCD porous polymers are shown in Figure 3. The average particle sizes of the PAA-MCTCD porous polymers were smaller than those of the LPEI-MCTCD porous polymers. High reactivity of the primary amine in the PAA side chain should accelerate the network formation rate, inducing fixation of the morphology at the early stage of phase separation. The reactions under the conditions of [MCT]/[NH] = 0.5 mol/mol and a lower materials concentration of 20 wt% (entries 9 and 12) formed a disordered morphology, likely derived from incomplete phase separation due to low phase separation rates. An increase in material concentration would accelerate phase separation, resulting in a morphology with connected particles (entries 10 and 13). In the reaction system of PAA1600-MCTCD, an increase in the [MCT]/[NH] molar ratio decreased the particle size in the resulting porous polymer (entry 11). The opposite result was observed in the PAA3000-MCTCD system (entry 14). One possible explanation for the result is a difference in preferential acceleration by the increase in [MCT]/[NH]—that is, the network formation rate in the PAA1600 system versus the phase separation rate.

The EPL-MCTCD system yielded porous polymers under reactions conducted at materials concentrations of 30 wt%. Increasing the [MCT]/[NH] feed ratio tended to decrease the particle size of the resulting porous polymer. An increase in crosslinking density should increase the network formation rate and fix the morphology at the early stage of the phase separation.

The standard deviation (SD) and coefficient of variation (CV) of the particle diameters are summarized in Table 1. The present porous polymers exhibited large CV values of more than 0.2. The LPEI500K-MCTCD porous polymer (entry 7: [MCT]/[NH] molar ratio of 0.5 in MeOH/H_2_O: 10 wt%/90 wt%) and PAA-MCTCD porous polymers (entries 9 and 12: [MCT]/[NH] molar ratio of 0.5) displayed disordered bulky structures with small, connected particles. The PAA3000-MCTCD and EPL-MCTCD porous polymers showed a bimodal particle size distribution (co-existence of small and large particles). One possible explanation for the wide distribution of sphere diameters is the coexistence of different concentrations in the reaction system, derived from phase equilibrium during the spinodal decomposition process, as previously reported in other reaction systems [31,32,33].

The specific surface area and pore size distribution of the porous polymers was evaluated by the Brunauer-Emmett-Teller (BET) and BJH methods. However, the surface area of all the samples was too small for quantitative evaluation. The low specific surface area may have been derived not from a “microporous,” but rather from a “macroporous” structure, as previously reported for other porous polymers.

### 3.3. Mechanical Properties of Porous Polymes

**Figure 5:**
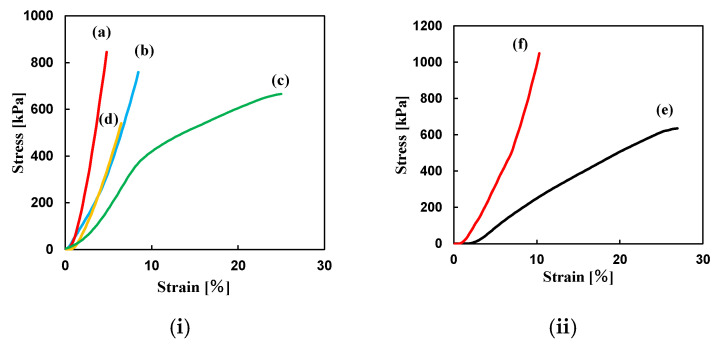
Stress–strain curves of (**i**) LPEI-MCTCD porous polymers: (a) LPEI50K (entry 1), (b) LPEI500K (entry 3), (c) LPEI50K (entry 5), and (d) LPEI500K (entry 7); and (**ii**) PAA-MCTCD porous polymer: (e) PAA1600 (entry 10) and (f) PAA3000 (entry 13).

The mechanical properties of the porous polymers, prepared under conditions of [MCT]/[NH]: 0.5 mol/mol and a materials concentration of 30 wt%, were investigated via the compression test. Figure 5 shows the stress–strain curves of the LPEI-MCTCD porous polymers. The structure and mechanical properties of representative porous polymers are summarized in Table 2. The LPEI500K-MCTCD porous polymers (entries 3 and 7) showed higher Young’s moduli than the LPEI500K-MCTCD porous polymers (entries 1 and 5). The bulk densities of the LPEI500K-MCTCD porous polymers were higher than those of the LPEI50K-MCTCD porous polymers. The small particle size in the LPEI500K-MCTCD porous polymers likely contributes to their high bulk densities. In the same way, the PAA3000-MCTCD porous polymer (entry 13) with higher bulk density showed a higher Young’s modulus than the PAA1600-MCTCD porous polymer (entry 10). All the porous polymers were broken under the compression of 50 N. The breaking strains of the porous polymers with higher Young’s moduli were less than 10%, indicating hard and brittle features. The stress–strain curves of porous polymers with low bulk densities (entries 5 and 10) exhibited two-step deformation. The initial hard and subsequent soft deformations might be derived from the compression of the particles and disintegration of the connected particles, respectively. A wide particle size distribution likely contributes to the fragility of these polymers.

The EPL-MCTCD porous polymers were too fragile to undergo mechanical property evaluation using the compression test.

#### Adsorption of Dyes

**Figure 6:**
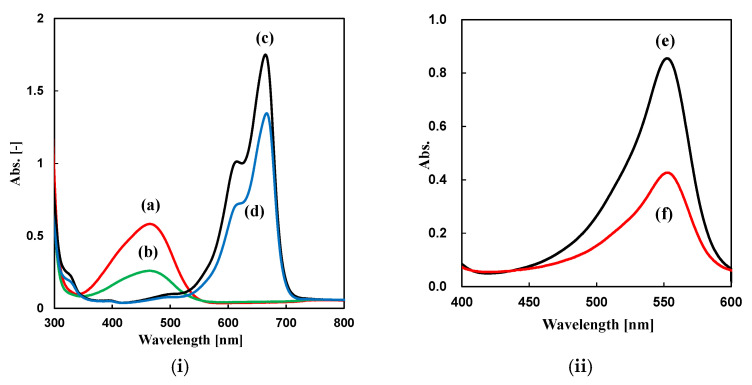
UV-Vis spectra of the (**i**) (a) original MO solution, (b) MO solution after the adsorption test, (c) original MB solution, (d) MB solution after the adsorption test, (**ii**) (e) original PP solution, and (f) PP solution after the adsorption test.

**Scheme 3:**
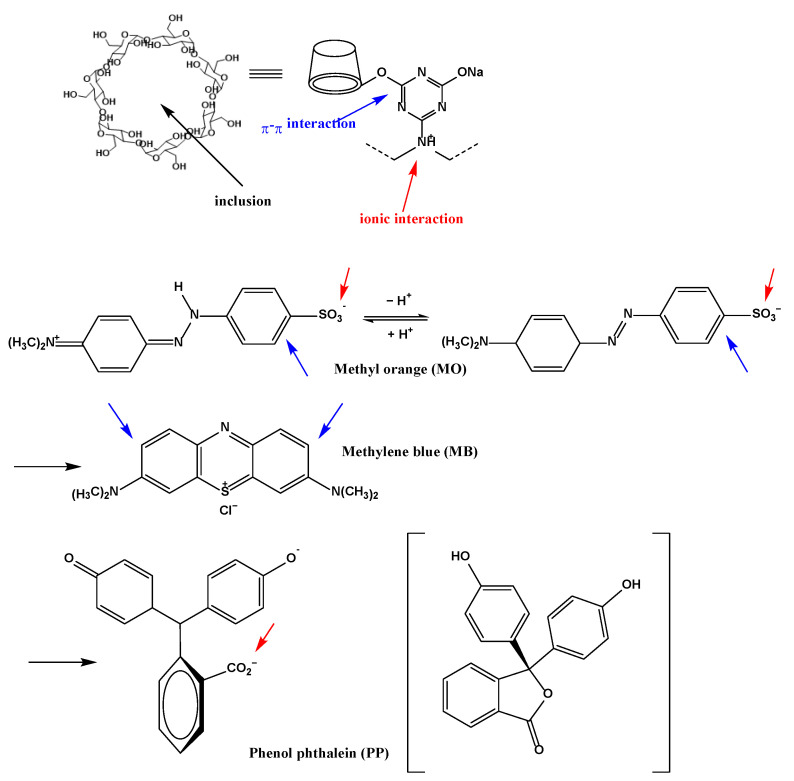
Structure of dyes and their plausible mechanisms of dye adsorption by LPEI50K-MCTCD porous polymer.

The present porous polymers have some molecular structures that can adsorb dyes. The macrocyclic structure of β-CD (in MCTCD), composed by seven glucose units with 0.6–0.8 nm holes, can include small organic molecules, especially with hydrophobic features. Gu et al. achieved efficient adsorption of dyes in aqueous solutions using sulfonated tetraphenylethylene-based hyper-crosslinked polymers. They reported that sulfonation of the polymers effectively induced ionic interactions with cationic dyes [34]. It may be possible to adsorb ionic molecules through ionic interactions between amine moieties in the porous polymers. Adsorption of MO by π–π interaction with cyclotriphosphazene moiety was previously reported [35]. Similarly, the triazin in the MCT moiety would also adsorb molecules through π–π interaction. These structures are suitable for the capture of organic compounds with the corresponding sizes, ionic moieties, and/or aromatic groups. In the present study, we tested the adsorption of dyes, MO, MB, and PP from the solutions by the LPEI50K-MCTCD porous polymer (entry 1). Figure 6 shows the UV spectra of the original dye solutions and those after immersion of the LPEI50K-MCTCD porous polymer. The decrease in peak intensities, attributed to the dyes, following immersion indicates their adsorption by the porous polymer. The possible adsorption mechanisms of MO, MB, and PP, along with the adsorption efficiencies evaluated from the peak intensities, are summarized in Scheme 3 and Table 3. Ionic interaction between the anionic moieties in MO or PP and cationic moieties of tertiary amines should induce the adsorption of these dyes. It is possible that π–π interaction may occur between the phenyl groups in MO and MB and the triazin ring in the MCT moiety. The macrocyclic structure β-CD can capture MB and PP. Hydrogen bonding may also contribute to dye adsorption. The adsorption efficiency of MO (50.9%) was higher than that of MB (24.9%) and PP (43.1%). The results indicate that the ionic interactions between the positively charged surface of the porous polymers and negatively charged dyes are effective for dye adsorption. In the present study, although selective adsorption of the dyes was not achieved, it may be possible to adsorb various molecules through multiple mechanisms derived from the characteristic molecular structures of the porous polymer.

The surface morphology of the LPEI50K-MCTCD porous polymer after MO adsorption was almost same as before the adoption test, indicating that dye adsorption occurs primarily on the surface and does not affect the morphology of the porous polymer.

## 4. Conclusions

The nucleophilic substitution reactions between linear polyamines (LPEI, PAA, and EPL) and MCTCD successfully yielded the corresponding porous polymers by simply mixing in water or MeOH/water. The porous morphologies were composed of connected particles, gathered particles, and/or disordered structures induced by phase separation via the spinodal decomposition process. The porous morphology was strongly affected by the polyamine, [MCT]/[NH] feed ratio, and materials concentration. The [MCT]/[NH] molar feed ratio of 0.5 and materials concentrations of 20–30 wt% were preferable for yielding the porous polymers. The LPEI-MCTCD and PAA-MCTCD porous polymers showed high Young’s moduli, which tended to increase with increases in bulk density. An LPEI-MCTCD porous polymer effectively adsorbed MO, MB, and PP by ionic interaction, π–π interaction, and/or β-CD inclusion.

Although the dye adsorption efficiency of the present porous polymers was not high, their molecular design may suggest a synergetic adsorption system for various organic molecules. As mentioned above, ionic interaction, π–π interaction, and β-CD inclusion can contribute to dye adsorption. The conditions for the adsorption, such as pH and solution polarity, can control the ionic and/or π–π interactions between the porous polymers and organic molecules. Furthermore, the introduction of α-CD or γ-CD into the porous polymers should enable size-selective adsorption of organic molecules. The porous polymers obtained from LPEI and PAA under the condition of an [MCT]/[NH] molar feed ratio of 0.5 contain unreacted NH groups. The structures can be used to capture carbon dioxide, formaldehyde, and other molecules. EPL is a biopolymer composed of L-lysine as a monomer. The combination of EPL and d β-CD can be used to produce functional porous polymers from biomass resources. Furthermore, antibacterial and preservative features derived from EPL can be added to EPL-MCTCD porous polymers. The high liquid and gas permeability of the porous structure is preferable for selective separation, adsorption, and/or sterilization. As a next step, we will apply the present types of porous polymer for these purposes, which can separate some molecules through chiral selective capture functionality. These investigations are currently underway, and the results will be reported elsewhere in due course.

## Figures and Tables

**Table 1:** Structure and properties of porous polyamine-MCTCD porous polymers.

Entry	Polyamine	Solvent (wt%/wt%)	[MCT]/[NH] mol/mol	Material Conc. wt%	Morphology ^1^	Average Diameter μm	SD ^2^	CV ^3^
1	LPEI50K	MeOH(20)/H_2_O(80)	0.5	30	C	6.03	1.44	0.24
2	LPEI50K	MeOH(20)/H_2_O(80)	1.0	30	C	3.98	0.80	0.20
3	LPEI500K	MeOH(20)/H_2_O(80)	0.5	30	C	4.78	0.93	0.19
4	LPEI500K	MeOH(20)/H_2_O(80)	1.0	30	C	3.38	0.96	0.28
5	LPEI50K	MeOH(10)/H_2_O(90)	0.5	30	C	4.87	0.97	0.20
6	LPEI50K	MeOH(10)/H_2_O(90)	1.0	30	C	3.46	0.62	0.18
7	LPEI500K	MeOH(10)/H_2_O(90)	0.5	30	D	n.d. ^4^		
8	LPEI500K	MeOH(10)/H_2_O(90)	1.0	30	C	3.29	0.10	0.30
9	PAA1600	H_2_O	0.5	20	D	n.d. ^4^		
10	PAA1600	H_2_O	0.5	30	G	2.95	0.40	0.14
11	PAA1600	H_2_O	1.0	30	D	n.d. ^4^	0.13	0.20
12	PAA3000	H_2_O	0.5	20	D	n.d. ^4^		
13	PAA3000	H_2_O	0.5	30	G	0.58	0.31	0.53
14	PAA3000	H_2_O	1.0	30	G	2.36	0.16	0.067
15	EPL	H_2_O	0.5	30	C	9.51	2.95	0.31
16	EPL	H_2_O	1.0	30	D	5.99	2.23	0.37

^1^ D: disordered structure, C: connected particles, G: gathered particles, Standard deviation of the diameter of the particles. ^2^ Coefficiency of varation. ^3^ SD/average diameter. ^4^ Not determined (n.d.) due to their distorted structures.

**Table 2:** Mechanical properties of the LPEI and PAA-MCTCD porous polymers.

Entry	Polyamine	Bulk Density	Young’s Modulus kPa	Breaking Stress kPa	Breaking Strain %
1	LPEI50K	0.384	3308	758.7	8.2
3	LPEI500K	0.421	3796	844.8	4.2
5	LPEI50K	0.279	831.8	807.2	36.4
7	LPEI500K	0.434	2103	539.7	5.6
10	PAA1600	0.315	318.4	649.6	26.4
13	PAA3000	0.389	1681	1049	9.3

**Table 3:** Adsorption tests of dyes from the solutions by LPEI50K-MCTCD porous polymer (entry 1).

Dye	Mechanism		Adsorption Efficiency %
MO	Ionic interaction	π-π interaction	50.9
MB	β-CD inclusion	π-π interaction	24.9
PP	Ionic interaction	β-CD inclusion	43.1

## Data Availability

The original contributions presented in this study are included in the article/Supplementary Materials. Further inquiries can be directed to the corresponding author(s).

## Supplementary Materials

The following supporting information can be downloaded at https://www.mdpi.com/article/10.3390/ma18112588/s1: Figure S1: Production diagram of LPEI-MCTCD systems, Figure S2: Production diagram of PAA-MCTCD systems, Figure S3: Production diagram of EPL-MCTCD system, Figure S4: FT-IR spectra of PAA-MCTCD system (entry 13), Figure S5: FT-IR spectra of EPL-MCTCD system (entry 15).
