# Supplementary material for: Synthesis of Porous Polymers by Nucleophilic Substitution Reaction of Polyamines and Monochlorotriazinyl-β-Cyclodextrin and Application to Dye Adsorption
Source: Materials (Basel). 2025 Jun 1;18(11):2588. doi: 10.3390/ma18112588 (PMC12155709; doi:10.3390/ma18112588)
Supplement: Supplementary file 1 [file materials-18-02588-s001.zip › materials-3626871-supplementary.pdf]

Supporting Information

Synthesis of porous polymers by nucleophilic substitution reaction of polyamines and monochlorotriazinyl-β-cyclodextrin and application to dye adsorption

Naofumi Naga [1,2*], Risa Hiura[2] and Tamaki Nakano [3,4]

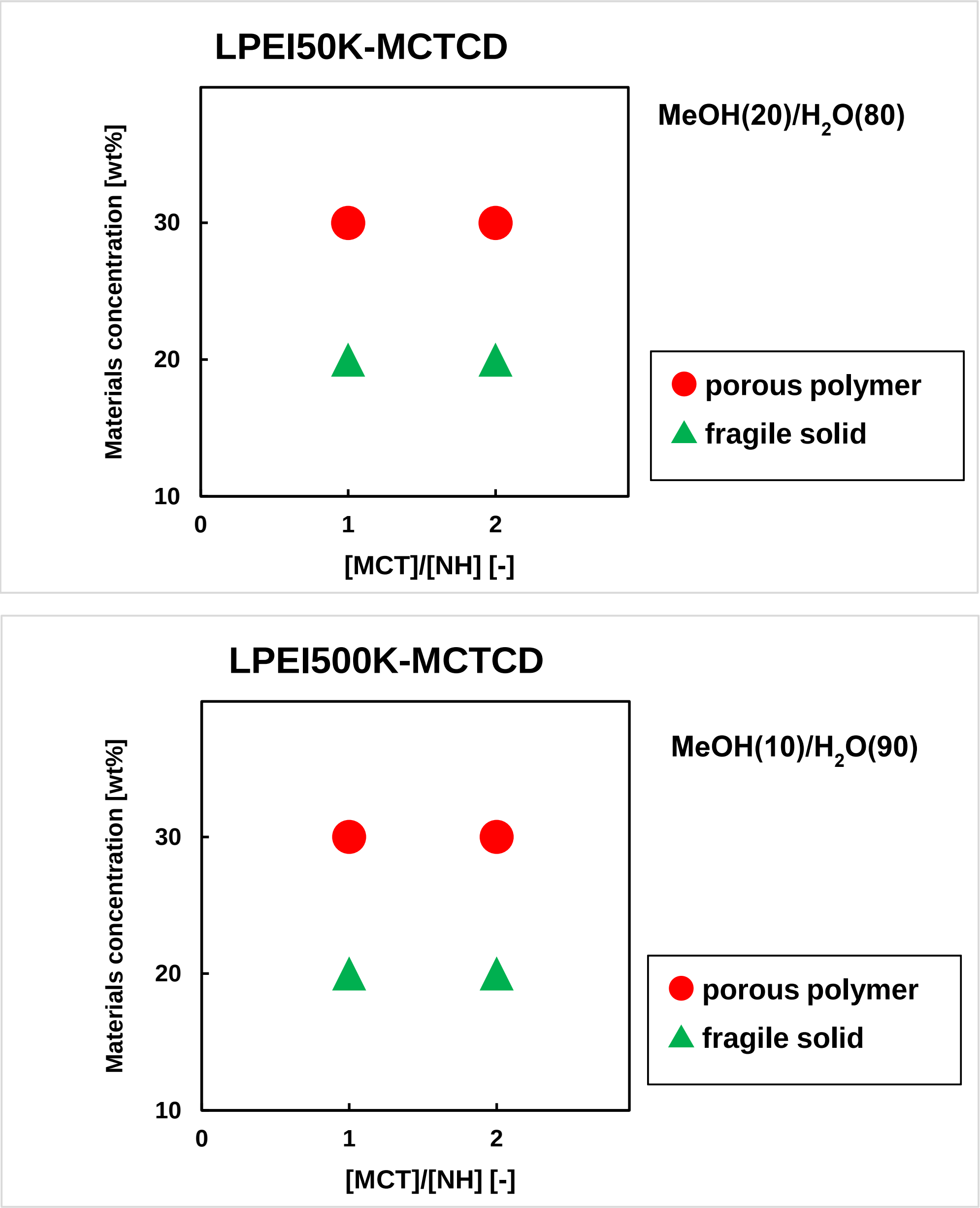

Figure S1. Production diagram of LPE-MCTCD systems.

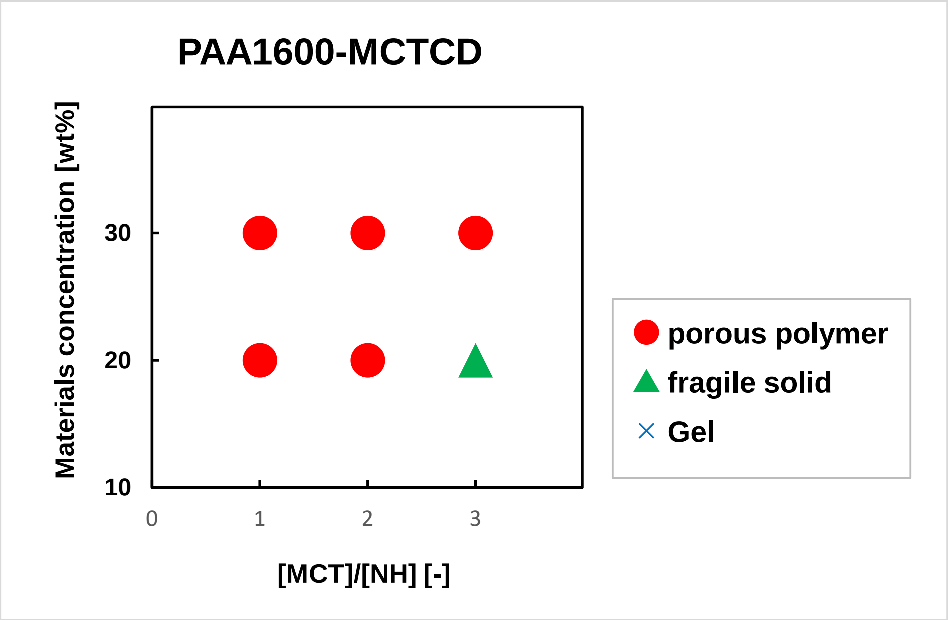
PAA1600-MCTCD
Materials concentration [wt%]
30
20
10
0
1
2
3
[MCT]/[NH] [-]
porous polymer
fragile solid
Gel

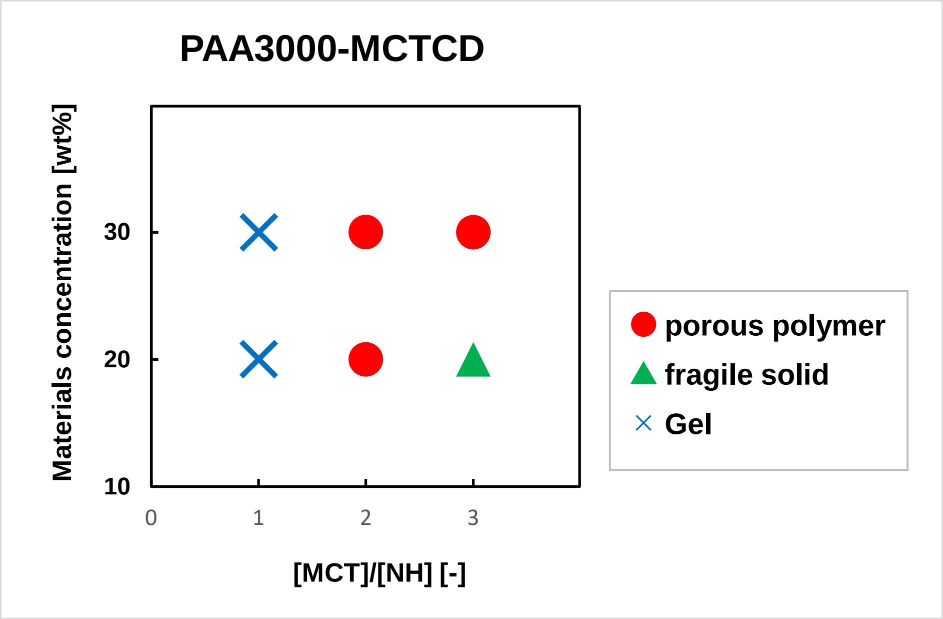
PAA3000-MCTCD
Materials concentration [wt%]
30
20
10
0
1
2
3
[MCT]/[NH] [-]
porous polymer
fragile solid
Gel

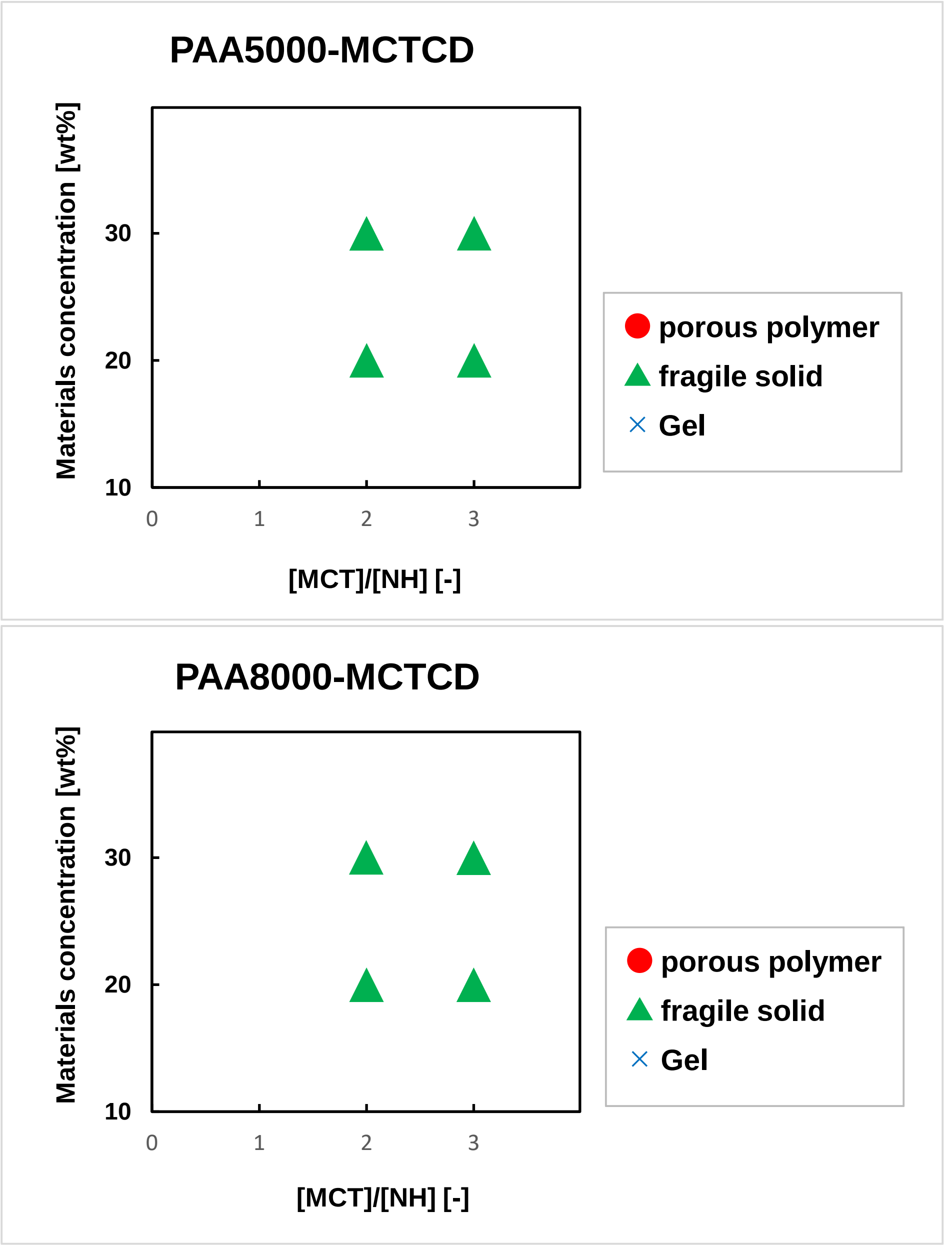

Figure S2. Production diagram of PAA-MCTCD systems.

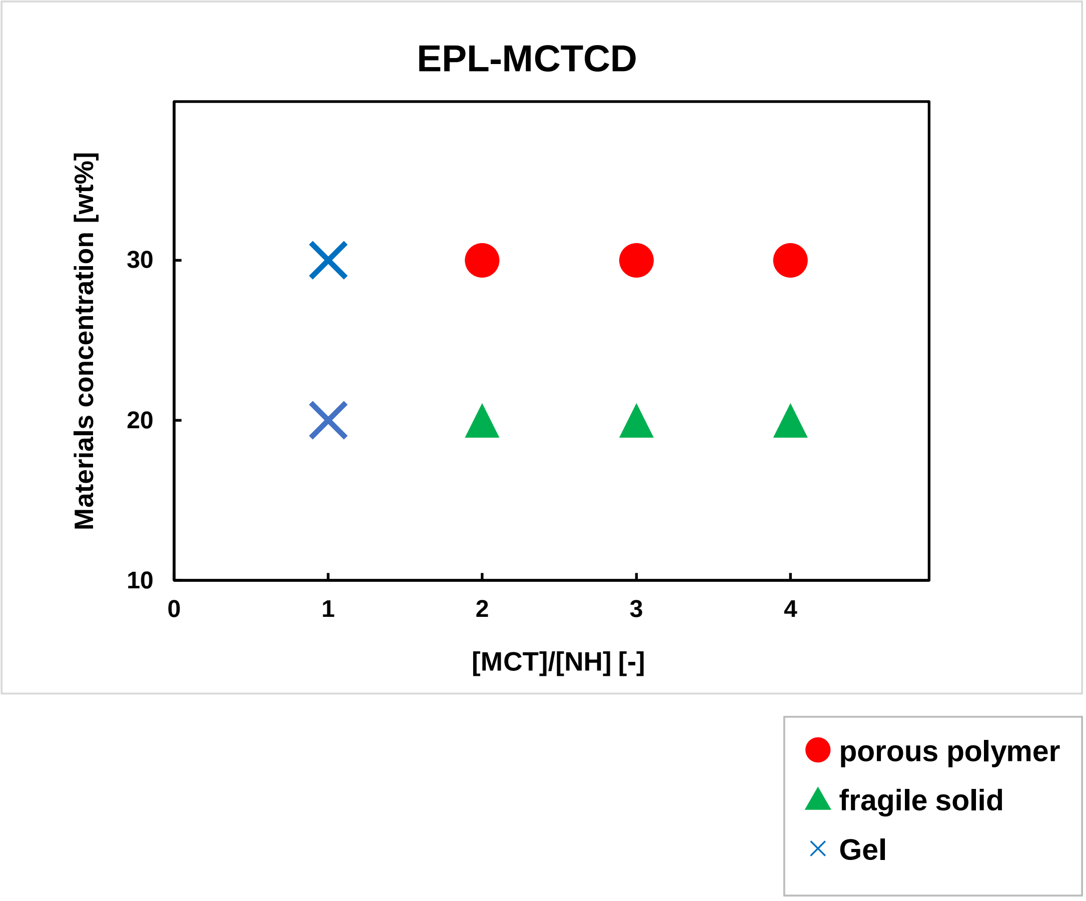

Figure S3. Production diagram of EPL-MCTCD system.

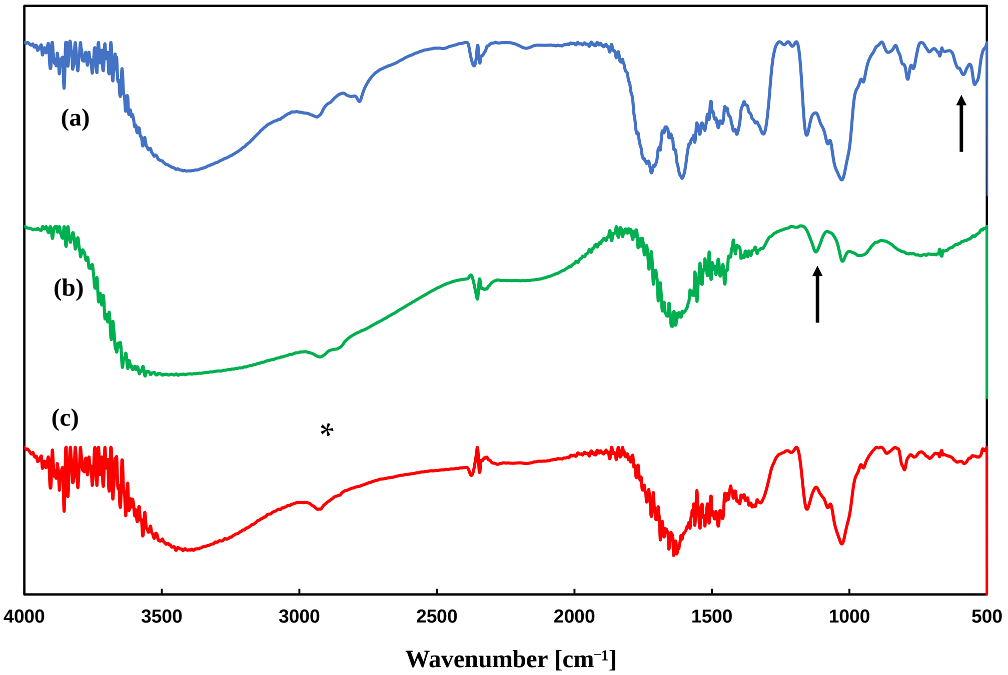

Figure S4. FT-IR spectra of PAA3000-MCTCD system, (a) MCTCD, (b) PAA3000, and (c) PAA3000-MCTCD, [MCT]/[NH] molar ratio: 0.5, materials concentration: 30 wt%, in water (entry 13).

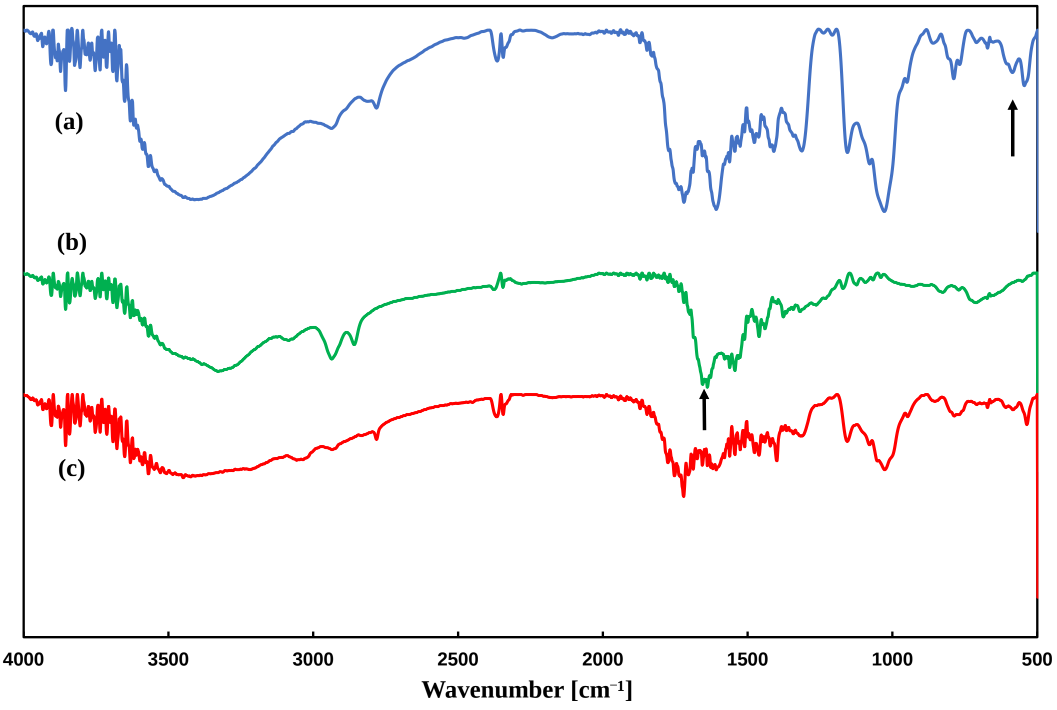

Figure S5. FT-IR spectra of EPL-MCTCD system, (a) MCTCD, (b) EPL, and (c) EPL-MCTCD, [MCT]/[NH] molar ratio: 0.5, materials concentration: 30 wt%, in water (entry 15).
